# Variation of the human mu-opioid receptor (OPRM1) gene predicts vulnerability to frustration

**DOI:** 10.1038/s41598-020-78783-4

**Published:** 2020-12-14

**Authors:** Alan M. Daniel, Brenda G. Rushing, Karla Y. Tapia Menchaca

**Affiliations:** Department of Science and Math, Texas A&M University-San Antonio, One University Way, San Antonio, TX 78224 USA

**Keywords:** Emotion, Human behaviour, Behavioural genetics

## Abstract

Understanding the emotional reaction to loss, or frustration, is a critical problem for the field of mental health. Animal models of loss have pointed to the opioid system as a nexus of frustration, physical pain, and substance abuse. However, few attempts have been made to connect the results of animal models of loss to human behavior. Allelic differences in the human mu opioid receptor gene, notably the A118G single nucleotide polymorphism, have been linked to individual differences in pain sensitivity, depressive symptoms, and reward processing. The present study explored the relationship between A118G and behavior in two frustrating tasks in humans. Results showed that carriers of the mutant G-allele were slower to recover behavior following a reward downshift and abandoned a frustrating task earlier than those without the mutation. Additionally, G-carriers were more sensitive to physical pain. These results highlight the overlap between frustration and pain, and suggest that genetic variation in opioid tone may contribute to individual differences in vulnerability and resilience following emotional disturbances.

## Introduction

Unexpected loss of reward results in an emotional reaction called *frustration*^1^. Frustration is most commonly studied using reward downshift procedures in which an individual is first trained to expect a large reward and is subsequently shifted to a lesser reward^2,3^. The surprising reward loss results in rejection of the new reward, followed by a recovery phase in which they come to accept the lesser reward. Studies with rats have demonstrated the involvement of several systems related to pain and stress during incentive downshifts. The downshift triggers HPA activation^4,5^, and the emotional response to the change in reward can be modulated by opioid drugs^6–9^. The discovery of shared mechanisms between pain and frustration^10,11^ in combination with comparative studies has led to the hypothesis that the capacity for frustration observed in mammals was coopted from the pain system early in the mammalian lineage^12^. Frustration from reward downshifts ties together the elements of reward processing, emotional disruption, opioids, and interaction with pain^10^.

Individual differences in sensitivity to the downshift and subsequent recovery serve as models of *vulnerability* and *resilience* in the face of loss^6^. Individual differences in recovery from reward downshift have been observed in rats^13^, with slow recovery rats exhibiting higher sensitivity to the nonselective opioid antagonist naloxone^6^. Artificial selection studies suggest that parental rates of recovery can have an impact on responses to reward downshifts in future generations^14^. Genes code for both the precursors for neuropeptides and for their receptors, and it is reasonable to posit that allelic differences in the opioid system may contribute to individual differences in responses to reward downshifts.

In humans there is a well-known variant of the mu opioid receptor gene (OPRM1) known as the A118G (rs1799971) single nucleotide polymorphism (SNP) of the mu opioid receptor (MOR), in which the typically occurring adenine at position 118 is replaced with guanine^15^. The change results in lower OPRM1 mRNA and reduced receptor proteins, suggesting that the SNP creates a defect in either transcription or mRNA maturation^16^. The prevalence of the mutation in the population is estimated between 10–32%, varying by ethnic group^17^. G-carriers have been shown to be more sensitive to physical pain and exhibit reduced effectiveness of opioid drugs in pain management^18–21^. The A118G mutation has been implicated in clinical depression risk^22^, stress responses^23–25^, high neuroticism^26^ and differences in reward processing^27,28^. There are also positive effects of the mutation, with reported increases in overall affect^29^ and social rewards^30,31^. Frustration is a negative emotion generated by reward comparisons that interacts with pain and is modulated by opioids, placing it at the center of these converging lines of research. Because of its prevalence and the links between pain and frustration, the A118G SNP is a likely target for genetic influence on rates of recovery following a reward downshift.

Given its ubiquity in daily life and the importance of frustration and its regulation to mood and anxiety disorders, there have been surprisingly few studies that target human frustration. The opioid system’s role in human reward loss and relative reward comparisons is unclear, but the picture that emerges from the human and animal studies reviewed above suggests a strong link between variability in opioid function and variability in recovery from reward loss. The present experiment explored the role of the A118G SNP in human pain and frustration. Two frustration tasks were administered, and physical pain was assessed via the cold pressor task. Consistent with previous research, we hypothesized that G-carriers would be more sensitive to physical pain in the cold pressor task compared to their AA homozygote counterparts. In the frustration tasks, we hypothesized that G-carriers would be more vulnerable to the reward downshift, exhibiting slower recovery or reduced persistence compared to the resilience of AA homozygotes.

## Materials and methods

### Participants

While many A118G studies target clinical populations (e.g., chronic pain, postpartum opioid treatment, substance abuse) there are important questions about the degree to which this genotype, which has high prevalence within the general population, is related to differences in behavior. Participants were recruited from a large participant pool managed by the psychology program, consisting of students enrolled in introductory level psychology courses or a genetics course for which there is a required research component. Participants chose this study from a list of opportunities to earn research credits required for their course. In order to be eligible, participants had to be at least 18 years of age. There were a total of 62 participants (53 females, 9 males) in the sample, which reflects the local demographics of the institution. All methods and procedures were approved by the Texas A&M University-San Antonio Institutional Review Board prior to the start of the experiment, and informed consent was obtained from all participants. All research was approved and performed in accordance with both institutional and U.S. regulatory guidelines under the Revised Common Rule.

### Materials and procedure

Participants came to an office suite that houses the psychology research laboratory between 12 and 6 pm, and were led to the data collection room. Data were collected in a quiet location at the back of the psychology research office suite, where only the researcher and the participant were present. Other people that came with the participant were asked to wait in the lobby, and the participant left their belongings at the front desk.

#### Reward downshift task

This task was used because it mirrors the incentive contrast procedures in nonhuman animals by training the individual to press a button for points. The behavioral tasks were administered via a 10″ Android tablet programmed using MIT App Inventor. Based on the Human Shuttle Box procedure^32^, four square targets were presented on a tablet screen at the top, bottom, left, and right. Participants were given instructions that did not describe the task but asked them to earn as many points as possible. One of the targets was randomly chosen to be a correct response, and each touch to that target yielded a message informing the participant that they earned points in the center of the screen. Touching the other targets had no effect. The correct target remained the same throughout the full procedure. After 10 s, the targets disappeared and the participant was notified of their point total for that trial. There was a 10 s intertrial interval (ITI) and then the targets reappeared. This continued for 18 trials. For the first 10 trials, participants received 32 points for each press, whereas during the 8 postshift trials each press yielded only 4 points. Once all 18 trials were complete the program automatically advanced to the next computer frustration task. The number of correct button presses was recorded for each trial.

#### Computer frustration CAPTCHA task

A second frustration task was designed based on the computer science literature, since frustration with human–machine interfaces is a critical problem to software design. In these studies, participants are typically given a computerized task, but the computer malfunctions causing a loss of reward^33^. While reward downshift studies are more similar to the rodent methods, the computer studies may better capture the meaning of frustration as it is used in common parlance. The question remains whether one or both of these approaches best fits the construct of frustration as it is understood in the context of animal models, and it is therefore valuable to explore both of these approaches.

Participants were told that to move on to the next phase they needed to first solve a Completely Automated Public Turing Test To Tell Computers and Humans Apart (CAPTCHA). These were selected from Google images as exceptionally difficult CAPTCHAs to solve. The program made them unsolvable by ignoring the participant’s typed input and presenting an error message. The participant had to then wait for 2 s before the program gave them a new CAPTCHA to solve. This continued for up to 20 unsolvable CAPTCHAs, or until the participant approached the researcher about having difficulty or that the program was not working at which point they were told to skip the rest of that phase. The number of CAPTCHAs attempted was the dependent variable.

#### Pain sensitivity

The Cold Pressor task was used to measure pain sensitivity. Participants were asked to immerse their non-dominant hand and forearm in a 40 × 32 × 15 cm plastic dishpan filled with finely ground ice and water to make a slushy mixture with a temperature of 5 ± 2 °C. Ice was replaced as needed between participants to maintain this temperature. Participants were asked to keep their hand submerged as long as they could, but they were told to remove their hand if it became too painful or uncomfortable. Previous studies have shown that the cold pressor task is nonmonotonic, with pain becoming replaced with feelings of numbness as time progresses^34^. For this reason, both subjective reports of pain intensity and hand removal latency were measured. The Wong–Baker faces pain scale^35^ was placed in front of the participant, with pain rating numbers ranging from 1 to 10. Once the participant placed their hand in the ice water, a stopwatch was started out of view of the participant. The participant was prompted to rate their pain every 15 s. Training continued until either the participant removed their hand or until there were three successive pain ratings reported with no change. A cap of 5 min was placed on the task, but no participants reached this criterion. The participant was then allowed to dry themselves off with a paper towel. Peak pain rating and latency to remove the hand were recorded.

#### Genotyping

Participants did not eat or drink during the half-hour prior to swabbing, since they were engaged in the behavioral tasks. Participants were asked to provide a DNA sample for genotyping using HydraFlock 6″ sterile swabs with handles (Puritan Medical Products, ME, USA). The participant swabbed the inside of the cheek for 10–15 s, then placed the swab inside an enclosed tube for short term storage at 4 °C. DNA was extracted using Zymo Quick-DNA Miniprep Kit (Zymo Research, CA, USA). Target DNA was then amplified by polymerase chain reaction (PCR) using previously published primers^36^ and the Q5 High-Fidelity 2 × Master Mix (New England Biolabs, Ipswich, MA). Amplification was confirmed by agarose gel electrophoresis and amplified DNA was purified from the PCR reactions using a Zymo Clean and Concentrate kit. Sequencing was outsourced (Sequetech, CA, USA). Sequencing traces were analyzed to determine presence or absence of the G alleles.

### Statistical methods

A number of statistical tests were employed based upon the properties of the data. Tests were chosen on the basis of assumptions that were or were not met; chiefly, tests of normality to determine whether to use parametric or nonparametric tests, and corrections for violations of the homogeneity of variance assumption were used when necessary. All tests were two-tailed with the level of α = 0.05, such that p-values less than 0.05 were considered statistically significant. Whenever appropriate, effect sizes were included as an indication of practical significance. All data were analyzed using the open source statistical analysis program JASP version 0.11.1 (University of Amsterdam, NH, NL).

Frequency data such as the distribution of males and females by genotype were analyzed using a Chi Square Test for Independence, with the effect size measure Cramer’s V. For ANOVAs comparing changes between groups across trials in the button-pressing task, the heterogeneity of variance assumption was tested using Mauchly’s sphericity test, and when the assumption was violated, a Huynh–Feldt sphericity correction was used to accommodate this result. Eta squared served as the estimate of effect size for ANOVAs. For comparisons between G-carriers and AA-homozygotes, a Shapiro–Wilk test of normality determined whether to use parametric or nonparametric tests. When both groups were normally distributed, the parametric Welch’s *t* test was calculated, and Cohen’s D was included as an effect size measure. The Welch’s *t* test was used because it does not assume homogeneity of variance but is still robust when the assumption is met, thus making it preferable in practice compared to the Student’s *t* test which assumes homogeneity of variance. For cases in which one or both groups was not normally distributed, the nonparametric equivalent Mann–Whitney *U* was used instead, with rank biserial correlation to indicate effect size.

Finally, since null hypothesis significance tests cannot provide support for null hypotheses, in the case that a null result was important to the interpretation of the data, a Bayesian equivalent of the test was calculated. Bayesian methods compare the likelihood of the observed data under the null hypothesis and the alternative hypothesis, and produce a Bayes Factor (BF) that is a ratio of these two numbers, providing a relative likelihood measure. BF greater than 1 indicates favor for the alternative, whereas BF less than 1 favors the null hypothesis. In this way, it can be shown that the data are more likely given the null hypothesis than the alternative.

## Results

Of the 62 participants, 35 were homozygous AA (AA), 21 were heterozygous AG, and 2 were homozygous GG for a total of 39% of participants carrying the G allele (G-carriers). For the remaining 4 participants, genotypes could not be determined and were excluded from statistical analyses. Because sex differences have been reported in pain sensitivity, distribution of males and females across genotypes were compared (Table 1). A Chi-square test for independence indicated that distribution of sex did not differ between AA and G-carriers, *χ*^2^(1, N = 58) = 0.178, *p* = 0.67; the effect size indicated little to no association, *Cramer’s V* = 0.06. This suggests that sex differences which may exist are balanced across genotypes, and are unlikely to account for differences observed between them (Table 2). Since this is a case in which the null result is important, a Bayes factor was computed comparing the data under the null and alternative hypotheses, *BF*_10_ = 0.44, suggesting that the null hypothesis was favored 2.29:1.

**Table 1:** Participant sex by genotype.

	Female	Male	Total
AA	29	6	35
AG	19	2	21
GG	1	1	2
Undetermined	4	0	4

**Table 2:** Behavioral task performance by sex and genotype.

Task	AA		G-carrier	
	*M*	*SD*	*M*	*SD*
**Recovery Index**				
Female	6.83	12.13	2.00	6.44
Male	16.50	9.61	− 2.00	10.44
Total	8.49	12.19	1.48	6.90
**CAPTCHA solved**				
Female	16.72	5.98	13.40	7.32
Male	17.67	5.72	9.67	9.07
Total	16.89	5.86	12.91	7.44
**Cold pressor time**				
Female	52.90	48.31	57.95	45.47
Male	46.00	27.96	27.33	15.37
Total	51.71	45.21	53.96	43.80
**Cold pressor rating**				
Female	6.59	2.35	7.95	1.54
Male	7.83	2.56	9.33	1.16
Total	6.80	2.40	8.13	1.55

### Reward downshift

**Figure 1:**
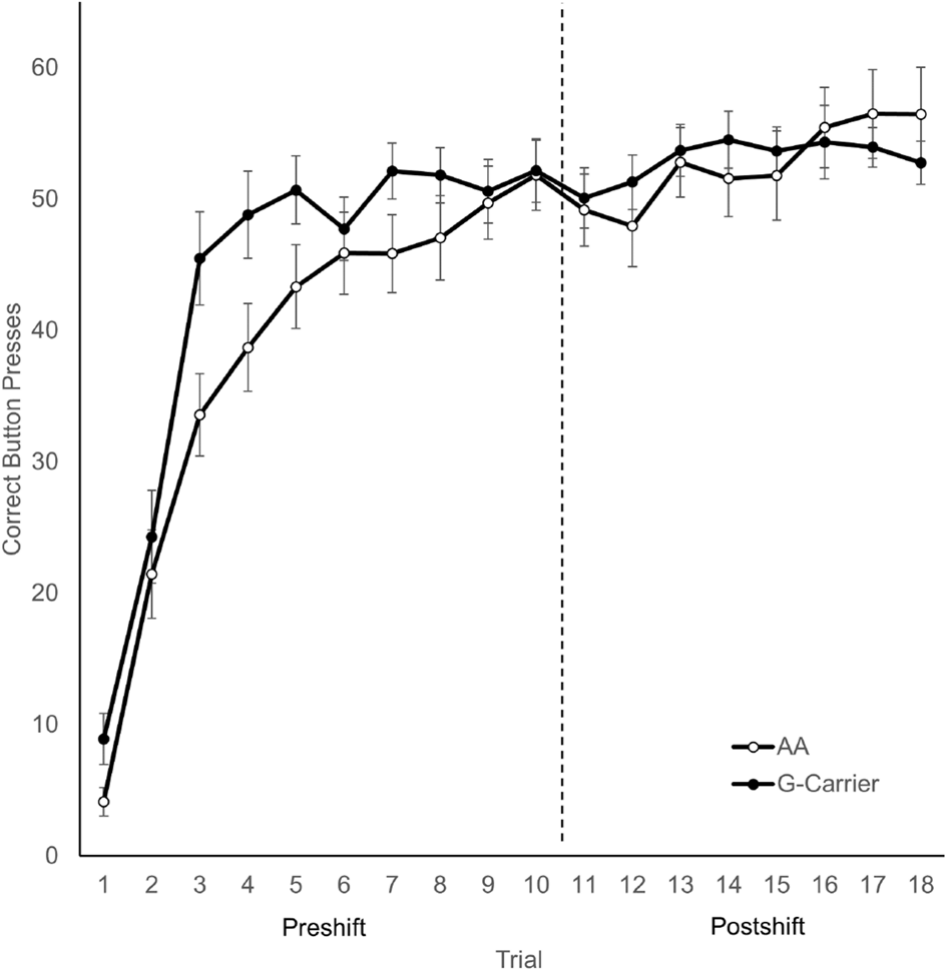
Average number of correct button presses in the reward downshift task (± SEM). During the preshift phase, G-Carriers exhibited faster acquisition of button pressing for a reward of 32 points per press. There was a significant overall trend for recovery of button pressing across the postshift phase.

Analyses were divided between the preshift phase, the postshift phase, and by an index of recovery rates. A 2 × 10 (Genotype × Trial) repeated measures ANOVA was conducted on the acquisition of correct button presses during the preshift (Fig. 1). Mauchly’s test indicated that the assumption of sphericity had been violated (*χ*^2^(44) = 214.67, p < 0.001); therefore degrees of freedom were corrected using Huynh–Feldt estimates of sphericity (ε = 0.60). Both groups acquired the button pressing behavior across trials, with a main effect of Trial, *F*(5.38, 302.27) = 134.05, *p* < 0.001, *η*^2^ = 0.42. A significant Genotype × Trial interaction suggests that G-carriers acquired faster than AA, *F*(5.40, 302.27) = 2.32, *p* = 0.04, *η*^2^ = 0.01. There was no significant main effect of Genotype, *F*(1, 56) = 2.125, *p* = 0.15.

The postshift phase was analyzed using a 2 × 8 (Genotype × Trial) repeated measures ANOVA (Fig. 1). Mauchly’s test indicated that the assumption of sphericity had been violated (*χ*^2^(27) = 47.50, *p* = 0.01); therefore degrees of freedom were corrected using Huynh–Feldt estimates of sphericity (ε = 0.91). Button pressing recovered across the postshift phase, as indicated by a main effect of Trial, *F*(6.38, 357.06) = 4.52, *p* < 0.001, *η*^2^ = 0.02. There was no effect of genotype, and no other effects or interactions were significant, *p*s > 0.05.

**Figure 2:**
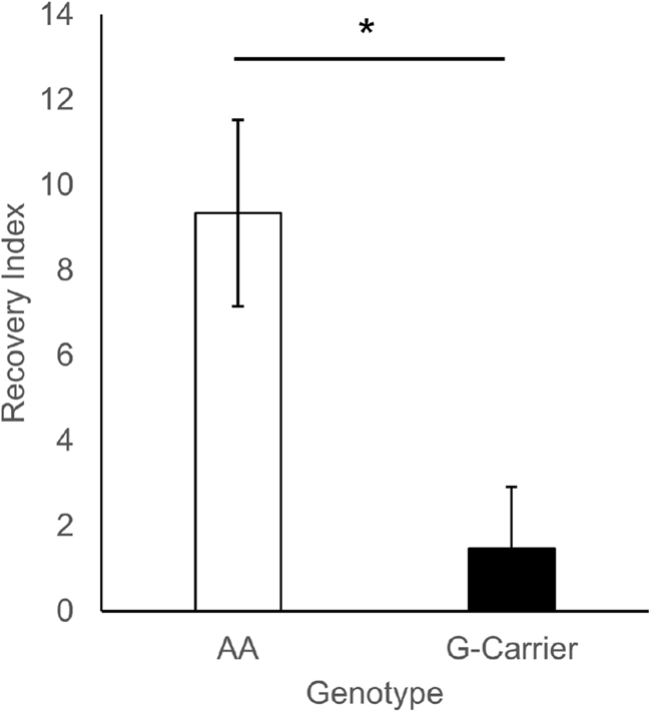
Average recovery index for each genotype (± SEM). AA recovered significantly faster than G-carriers.

A recovery index was used to distinguish between high and low recovery individuals in the sample, calculated as a difference score between Trial 12 (during the shift) and Trial 18 (full recovery, Fig. 2). Trial 12 is the first trial following the first postshift trial score feedback, and for this reason, it is a better indicator of the reaction to the downshift than responses on Trial 11. A Welch’s *t* test confirmed that there was a significant difference in the index of recovery between the genotypes, with G-carriers exhibiting lower recovery across the postshift period, *t*(55.03) = 2.79, *p* = 0.007. The effect size was medium, *Cohen’s D* = 0.71.

### CAPTCHA

**Figure 3:**
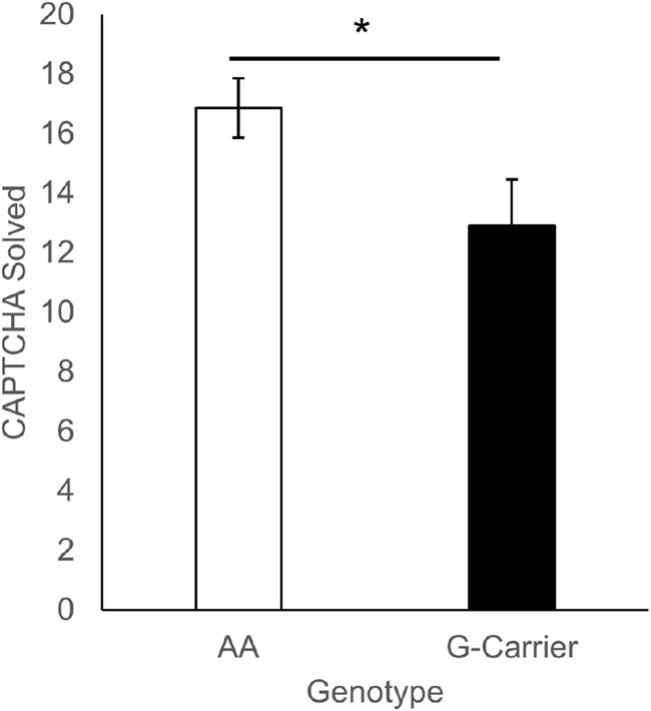
Number of CAPTCHA images attempted by genotype (± SEM). G-carriers abandoned the task significantly earlier than AA.

The responses in the CAPTCHA task were not normally distributed, since many participants either quit early on or went all the way to the maximum, therefore a Mann–Whitney nonparametric test was used for analysis. If G-carriers were more sensitive to the frustration of the task, it would be expected that they would quit earlier than AA (Fig. 3). G-carriers were, in fact, more likely to abandon the task early (*M* = 12.9) compared to AA (*M* = 16.9), *U* = 516.5, *p* = 0.036, rank biserial correlation = 0.28.

### Cold pressor

**Figure 4:**
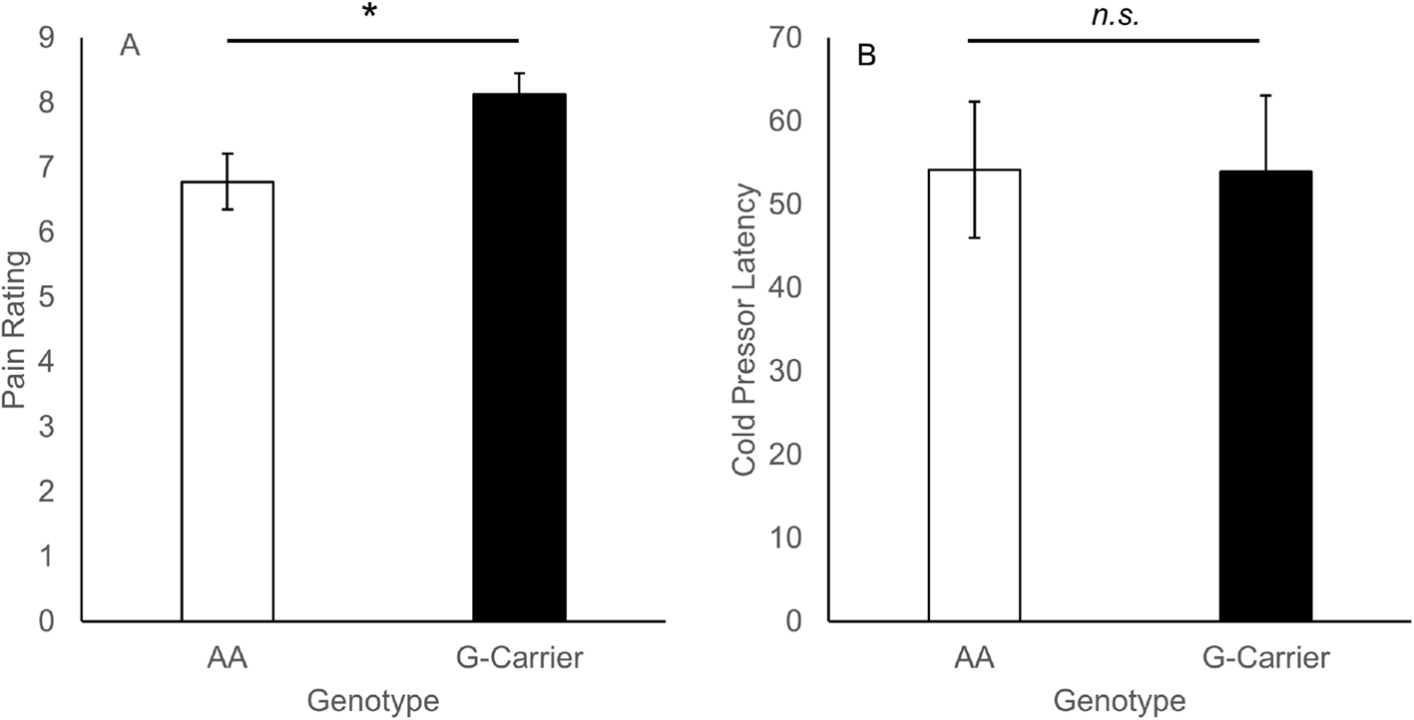
Pain ratings (**A**) and latency to remove the hand (**B**) in the cold pressor task (± SEM). G-carriers rated the task as significantly more painful than AA, though hand-removal latency did not differ.

G-carriers rated the cold-pressor task as significantly more painful compared to AA (Fig. 4), supported by a Mann–Whitney *U* = 271.5, *p* = 0.036. The effect size was medium, rank biserial correlation = 0.33. There was no significant difference detected between genotypes in hand removal latency, *U* = 391.5, *p* = 0.87, rank biserial correlation = 0.03. No participants reached the 5 min maximum latency.

## Discussion

The opioid system is central to pain, frustration, mood disorders, and substance abuse. Discovering genetic risk markers for vulnerability and resilience in the face of reward loss may lead to better ways to manage risk and suggest different treatment pathways, especially for individuals who are nonresponsive to the current popular treatment options. The present exploration into the widely prevalent A118G mutation of the opioid system was the first to link genetic variation in human opioid function to a behavioral assay of reward loss. The data presented here suggest that G-carriers exhibit vulnerability in frustration, characterized by less persistence in a frustrating task and lower recovery from a reward loss compared to AA heterozygotes. This is consistent with rat studies in which individual differences in opioid function are associated with differences in recovery rates^6^, as well as human studies that show G-carriers are more prone to depressive symptoms following life events involving social loss^22^. Furthermore, G-carriers in the present experiment were more sensitive to physical pain, as they rated their pain higher in the cold pressor task. The present results highlight a common opioid mechanism between pain and frustration found in animal models, observed here for the first time in humans. The increased frustration observed in G-carriers adds to a growing literature that characterizes the A118G phenotype as having an altered opioid tone that leads to the common outcomes of differences in emotion regulation, pain sensitivity, and reward processing. Individual differences in opioid tone across the general population which are, in part, genetically determined may play a significant role in emotional responses to day-to-day frustrating stressors.

There are a number of limitations with the present study that should be addressed. First, the present study was limited to a relatively low sample size. While sample sizes are substantial for this type of task as measured in animals, human and genetic studies of individual differences tend to require larger sample sizes to obtain sufficient power. As such, these data should be taken as a preliminary step toward linking opioid function to laboratory assays of reward loss; the promising results reported here should encourage replication. Additionally, the available participant pool was disproportionately female. There is evidence that there are nuanced sex differences in pain sensitivity and pain perception^37^, with a trend to observe greater pain sensitivity in women compared to men. In the present study males and females were proportionally balanced across genotypes which should minimize this issue. Ostensibly, these differences would increase variability in the data, serving to obscure the observed differences between genotypes. Now that the effect has been established, it should be replicated and extended to further explore the role of other known modulating factors such as sex, hormonal changes (e.g., menstrual cycles), mood, circadian effects, social variables, and so on, which will further elucidate the conditions under which the effects reported here are observed. The behavioral tasks used in the present study are simple to administer and could easily be adapted to be given remotely. More studies using and adapting these tasks may lead to the development of a simple, low-risk behavioral assay to identify risk factors for serious issues related to mood disorders, pain, and substance abuse risk.

In the cold pressor task, G-carriers were more sensitive than AA homozygotes, but only with regard to the peak intensity reported and not in terms of hand removal latency. There are a number of possibilities why this is the case. First, static measures of pain intensity and duration are processed differently^38,39^, which may account for this difference. Second, the cold pressor task tends to be nonmonotonic, such that pain may be replaced with feelings of numbness after a few minutes^34^. Participants who were able to continue past their peak pain rating may have experienced this change, which would obscure differences between genotypes. Because the latency measure was relatively unconstrained, there were substantial individual differences observed which obfuscated any between-subjects effects. Disentangling specific components of the dynamic pain process, such as anticipation, summation, and after-sensations may be useful to better illuminate how the A118G mutation impacts pain perception.

Another issue that warrants further exploration is the relationship between the two frustration tasks. Both tasks presumably involve a violation of the participant’s expectations, thereby inducing frustration. The CAPTCHA task is novel to the present experiment, and therefore this is the first step toward characterizing it as a measure of frustration as conceptualized in the reward downshift literature. The observed result shows that the G-carriers abandon the task in such a way that is consistent with the frustration explanation. Additionally, the subjective experiences spontaneously reported by participants while solving CAPTCHAs suggest aversion to the task; with comments such as “Oh my god, I hate these things!” and “Is this a joke? This has to be a joke.” However, it is possible that the task reflects another motivational process that promotes persistence without generating frustration. While the CAPTCHA task has face validity and appears to be under the influence of OPRM1 like the reward downshift task, further evidence is needed to characterize CAPTCHA as a frustration measure.

### Future directions

There are a number of areas ripe for exploration, both in terms of the behavioral tasks and the A118G mutation along with other genes important to opioid function. Recently, we demonstrated that the A118G mutation does not account for individual differences observed in rat recovery from reward downshifts^40^, suggesting there may be other important genetic influences on reward downshift that are as yet unknown. Bottom-up approaches such as a genome wide association study or more targeted efforts toward genes established to impact other types of emotional or reward processing tasks may elucidate some of these mechanisms.

Based upon the pattern of results observed here, we also suggest that opioid tone may impact reward processing in different ways. While G-carriers appeared more sensitive to frustration, they also appeared to be more sensitive to appetitive rewards as evidenced by faster acquisition of button pressing; two results that might seem at odds. Rewards have a value in the real world that must be represented in the brain which likely has an associated psychometric function. Psychometric functions relate task performance to an independent variable, reward value in this case, and are frequently used to assess sensitivity to perceptual stimuli. The psychometric function, the subjective scale of valences against which a target stimulus is compared, may have multiple features. For example, morphine administration might make rewards more positive and pain less negative, shifting the whole psychometric function to make all rewards more positively valenced. However, there’s also the possibility that differences in opioid tone such as those observed in the present experiment might impact the *slope* of the function. In such a case, individuals with a steeper slope would be more sensitive in both directions, with positively valenced stimuli appearing better and negatively valenced stimuli seeming worse. To test this, a psychometric curve could be established for a variety of reward (or reward downshift) values, then opioid treatments or variations in opioid function could be compared to see how the curve responds. This would provide a better understanding of how opioid tone, functional opioid differences, and opioid treatments impact the relative valences of stimuli which might apply across the domains of pain, reward, addiction, and emotion.

There is a strong need to understand human frustration and its underlying mechanisms, and connect the rich animal literature on incentive contrast to the human animal. Our results support a link between genetically modulated endogenous opioid activity and individual differences in recovery from reward downshifts in humans. Frustration may be an important element in understanding the links between opioid function, pain, addiction, and mental illness.
